# Hansen’s Disease, the Perpetual Impersonator (“The Behroopiya”) of Rheumatology Practice: A Case Series

**DOI:** 10.31138/mjr.310823.hdt

**Published:** 2023-08-31

**Authors:** Kavita Krishna, Sandeep Sadashivrao Kansurkar, Akanksha Kapoor, Sri Lakshmi Sathiyaseelan, Deepti Agarwal

**Affiliations:** Department of Clinical Immunology and Rheumatology, Bharati Vidyapeeth Medical College and Hospital, Pune, India

**Keywords:** Hansens, rheumatology mimics, infectious mimics, mimics of connective tissue diseases, mimics of Rheumatoid Arthritis

## Abstract

We encountered five patients with Rheumatic symptoms, suspected to have five different clinical diagnoses: Systemic vasculitis, Gout, Systemic Sclerosis, Rheumatoid Arthritis, and Spondyloarthropathy. However, all were finally diagnosed with the same illness: Hansen’s Disease.

## INTRODUCTION

**Figure 1.**
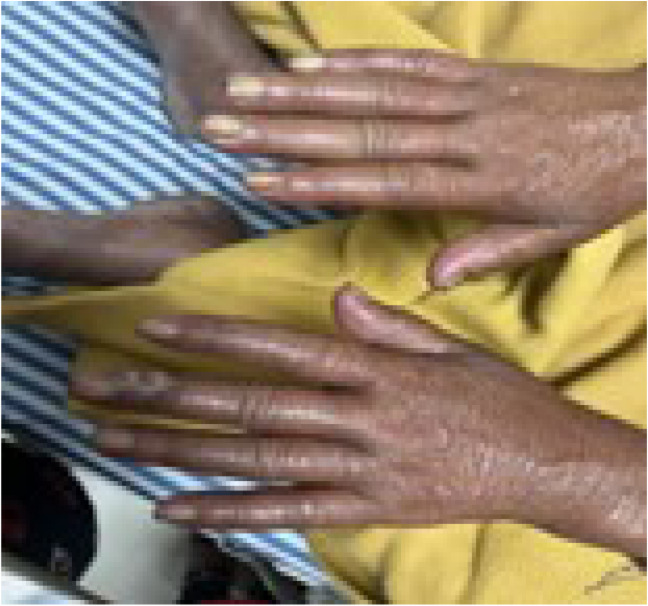
Case 1: Symmetric polyarthritis with a painless cutaneous ulcer over the right middle finger.

Mimicry has always existed: in biology, it was a process of evolution, within or among different species of organisms, often an anti-predator adaptation. However, mimics in Medicine and Rheumatology impel the diagnosticians to shed off their security blanket, visit, and revisit the scenario, looking for new clues, until they get it right. In 2005, the World Health Organisation (WHO) declared leprosy as an “eliminated” global public health problem, but over 200,000 new cases are recorded each year all over the world. The free treatment program by WHO is often underutilised, perhaps due to the stigma associated with the disease.^1^ For a physician and rheumatologist, clinical and serological similarities between patients with Hansen’s Disease (HD) and connective tissue disorders can lead to erroneous or delayed diagnosis. From May 2022 to January 2023, we encountered five interesting cases who visited the Rheumatology clinic with different clinical presentations mimicking rheumato-logical diseases, but in all these cases, we finally arrived at the same diagnosis: Hansen’s Disease. We collected clinical data from all these cases and clinical images were taken (**Figure 1**). Written informed consent was obtained from all patients. We aim to describe the clinical details of these cases. Clinical images are depicted in **Figure 1**.

## CASE DESCRIPTIONS

### Case 1

A 47-year-old lady presented with inflammatory polyarthritis involving small joints of the hands symmetrically for the past 6 months. She also had rash and induration over the face and trunk (**Figure 1**) along with a loss of temperature and pain sensation over her hands and feet for the past 1 month. She had developed multiple ulcers over her hands and feet for the past 15 days. She had been initiated on a disease-modifying anti-rheumatic drug (methotrexate 15 mg/week) and low-dose steroids 3 months ago. Because of lack of response, she omitted the medications after 1 month. On examination, she appeared pale, and had multiple neuropathic ulcers, especially over the great toe of her right foot. There was a hypoaesthetic facial rash along with an indurated skin over the nose. Bilaterally thickened ulnar nerves could be palpated. There was loss of temperature sensations over the palms and soles. On evaluation, a split skin smear (SSS) revealed granulomatous inflammation centred around neurovascular bundles suggestive of borderline lepromatous leprosy. Multi-drug therapy (MDT) was started, and she reported significant improvement in her symptoms on follow-up visits.

### Case 2

**Figure 2.**
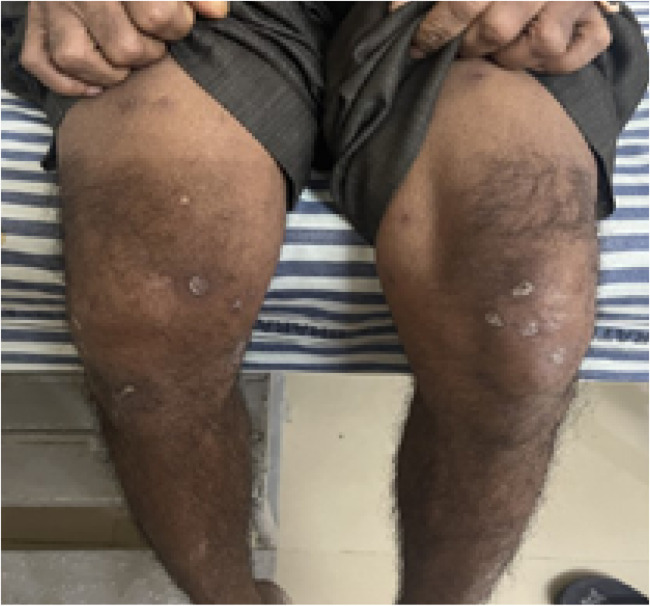
Case 2: Multiple erythematous non-pruritic papular lesions over the arms and knees.

A 59-year-old diabetic, hypertensive man with chronic kidney disease presented with an acute episode of severe pain and swelling in the left ankle, knee, and elbow joint. Inflammatory markers were elevated with hyperuricemia and sonographic evidence of crystal arthropathy. The patient was diagnosed with gouty arthritis and was treated for the same. Four months later, he presented with rash- multiple erythematous non-pruritic papular lesions over the arms, knees, and earlobes (**Figure 2**). Lesions had appeared over a 15-day period and were associated with high-grade fever. Dermatologist was consulted. SSS showed AFB positivity. Furthermore, the skin biopsy suggested tuberculoid granulomatous inflammation. Diagnosis of Erythema Nodosum Leprosum secondary to Hansen’s disease was made and 1mg per kg glucocorticoids were started. He showed dramatic improvement of his skin rash and joint pain following which he was started on MDT for Paucibacillary leprosy.

### Case 3

**Figure 3.**
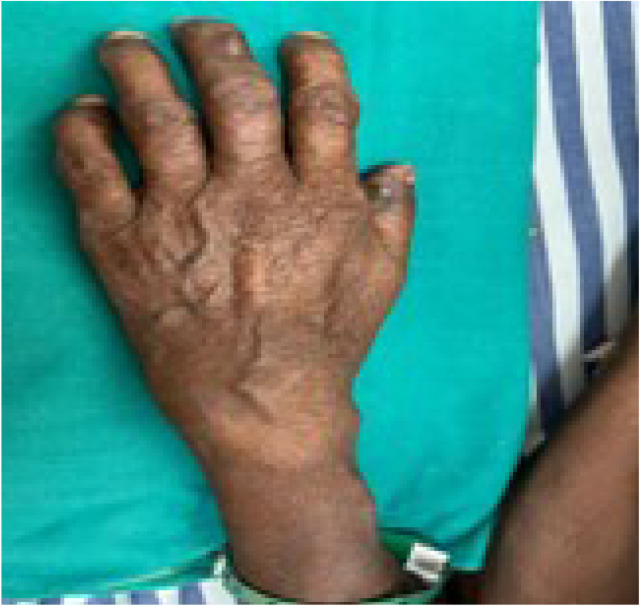
Case 3: Skin thickening, hand deformities, and multiple hypopigmented hypoaesthetic patches.

A 65-year-old lady was admitted with acid reflux symptoms for upper GI endoscopy. She was referred to the rheumatology department for skin tightening, Raynaud’s phenomenon, multiple digital gangrene to rule out a connective tissue disease. She had multiple joint deformities and the ulnar nerve appeared thickened bilaterally (**Figure 3**). A dermatology opinion was sought. The SSS revealed Lepromatous Leprosy with AFB positivity. Accordingly, a 1-year course of MDT was commenced which resulted in significant improvement in her symptoms.

### Case 4

**Figure 4.**
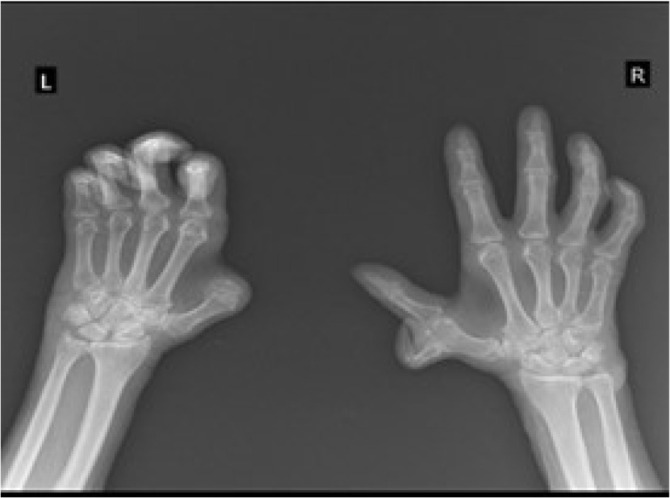
Case 4: Left > Right hand deformities.

A male aged 78 years was referred to the rheumatology department in view of multiple hand deformities for 5 years. However, he had never been evaluated for the same. He had a left claw hand deformity and the skin over his nose appeared infiltrated with a loss of eyebrows laterally. He also had thickened ulnar nerve (**Figure 4**). SSS was ordered which revealed AFB positivity. Subsequently, he was started on MDT.

### Case 5

**Figure 5.**
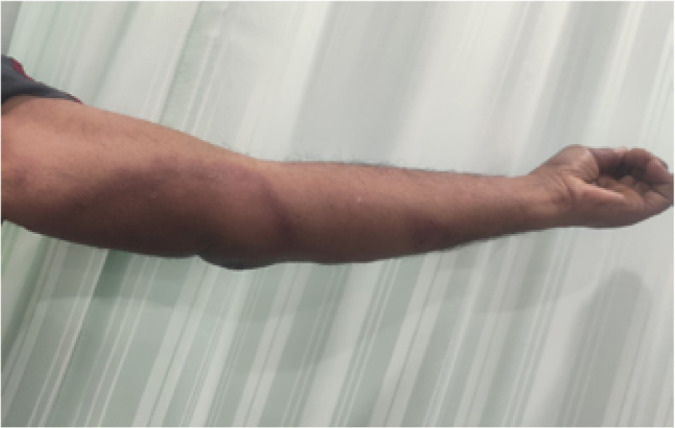
Case 5: Erythema Leprosum Nodosum.

A 63-year-old man presented to the orthopaedics department with a complaint of low backache for 1 year. He had a history of pain and swelling of small joints of the hands for the past 2 years. There was L4-L5 spondylolisthesis on imaging and was posted for a corrective procedure for the same. Meanwhile, he developed an erythematous maculopapular rash over his left forearm (**Figure 5**). Dermatology advised a SSS and Skin Biopsy which were consistent with the diagnosis of erythema nodosum. Additionally, on evaluation for his inflammatory polyarthritis, he was found to have rheumatoid factor and anti-cyclic citrullinated positivity. X-ray of the hands showed multiple erosions in left hand joints. He was started on 1 mg/kg prednisolone with gradual tapering, along with MDT. His Lepra reaction resolved over the next 6 months and joint symptoms had also reduced.

## DISCUSSION

As seen in this case series, apart from the well-known neurological and cutaneous manifestations of HD, musculoskeletal features are also commonly encountered. Musculoskeletal manifestations of Hansen’s disease include a varied pattern of arthritis, and one study has reported a prevalence of 54.28% in leprosy patients, and leprosy can even present with arthritis as the first symptom.^2^ But there is no formal classification for the musculoskeletal manifestations despite being the third most common symptom of HD. Henriques et al. have suggested a classification for arthritis in leprosy; acute symmetrical arthritis of lepra reaction and chronic arthritis.^3^ Chronic arthritis is an entity with rheumatoid arthritis‐ like distribution and features. Various autoantibodies are seen with HD – ANA (0-37.5%), RF (16–41%), antiCCP (5–9%).^4^ This confuses the distinction whether they are 2 separate processes or one. Given that anti-CCP is known to be the most specific antibody for RA and that chronic inflammation and advanced age can cause patients to produce nonspecific antibodies like ANA and RF, it is conceivable that these patients have chronic arthritis due to HD with the production of confounding nonspecific antibodies. The other facet of HD is that it could actually coexist with RA, as in our Case 5. Thus, it may be necessary to treat both the inflammation and the infection.^5^ Arthritis associated with Hansens disease coexisting with RA was seen in 21% cases in a study.^6^ In an another series, 10% of cases developed RA after resolution of HD.^7^ The pathogenesis of this occurrence is not fully understood but a number of hypotheses have been put forward.^8^ Accurate clinical diagnosis and X-Rays remain the key. As for our young students and Rheumatologists to-be, one can only re-emphasize that rheumatology is strongly rooted in internal medicine. In the era of super-specialities and sub-specialities, high-tech investigations, one should not undermine the importance of a comprehensive assessment - detailed history taking, meticulous clinical examination of the entire body (including looking for thickened nerves) and correlation with investigations. A high index of suspicion is imperative in patients with arthritis, positive RF, skin lesions, and peripheral neuropathy: or we may just board the wrong bus.
